# Robot-assisted total laparoscopic pancreaticoduodenectomy with transvaginal specimen extraction: a case report

**DOI:** 10.1093/jscr/rjag909

**Published:** 2026-10-10

**Authors:** Chiying Xu, Lei Lin, Kun Yang, Guanlei Liu, Taiyuan Li, Jinyuan Duan

**Affiliations:** Department of General Surgery, The First Affiliated Hospital, Jiangxi Medical College, Nanchang University, No. 1519 Dongyue Avenue, Nanchang County, Jiangxi Province, China; Department of General Surgery, The First Affiliated Hospital, Jiangxi Medical College, Nanchang University, No. 1519 Dongyue Avenue, Nanchang County, Jiangxi Province, China; Department of General Surgery, The First Affiliated Hospital, Jiangxi Medical College, Nanchang University, No. 1519 Dongyue Avenue, Nanchang County, Jiangxi Province, China; Department of General Surgery, The First Affiliated Hospital, Jiangxi Medical College, Nanchang University, No. 1519 Dongyue Avenue, Nanchang County, Jiangxi Province, China; Department of General Surgery, The First Affiliated Hospital, Jiangxi Medical College, Nanchang University, No. 1519 Dongyue Avenue, Nanchang County, Jiangxi Province, China; Department of General Surgery, The First Affiliated Hospital, Jiangxi Medical College, Nanchang University, No. 1519 Dongyue Avenue, Nanchang County, Jiangxi Province, China

**Keywords:** natural orifice specimen extraction surgery (NOSES), pancreaticoduodenectomy, robotic surgery, transvaginal, case report

## Abstract

Natural orifice specimen extraction surgery reduces incision-related complications and accelerates postoperative recovery while maintaining oncologic safety, whereas the da Vinci robotic surgical system further enhances the precision of minimally invasive procedures. This case report describes a robot-assisted total laparoscopic pancreaticoduodenectomy combined with transvaginal specimen extraction (RPD-TVSE). The patient was a 64-year-old female with moderately differentiated adenocarcinoma of the descending duodenum. The procedure took 350 min, with intraoperative blood loss of 200 mL; no blood transfusion was administered during the perioperative period. The patient was able to ambulate on postoperative day 1, with no adverse events recorded. Histopathology confirmed tumor staging as pT2N0M0, with all surgical margins negative. At 12-month follow-up, no evidence of recurrence was observed. This case demonstrates that RPD-TVSE is technically feasible and yields favorable short-term outcomes, providing a novel minimally invasive surgical option for female patients. However, long-term oncologic safety requires further investigation and validation.

## Introduction

Laparoscopic pancreaticoduodenectomy (LPD) has been demonstrated to be safe and feasible, with short-term outcomes comparable to those of open pancreaticoduodenectomy (PD) [1]. Nevertheless, inherent limitations of conventional straight-stick laparoscopic instruments—including restricted range of motion, two-dimensional visualization, and an unstable camera platform—pose substantial challenges during critical steps such as fine vascular dissection and pancreaticoenteric anastomoses.

The robotic surgical system addresses these limitations by providing three-dimensional stereoscopic vision, articulating wristed instruments, and tremor filtration, enhancing suturing precision in deep operative fields [2]. Natural orifice specimen extraction surgery (NOSES) enables specimen retrieval through a natural orifice, minimizing abdominal wall trauma and reducing incision-related complications [3]. Transvaginal specimen extraction (TVSE) is a well-established NOSES route in female patients [4]. While laparoscopic PD-TVSE has been reported, the robotic approach offers distinct advantages in performing precise intracorporeal anastomoses and navigating the pelvic cavity during transvaginal extraction. We present a case of robot-assisted total laparoscopic LPD with transvaginal specimen extraction (RPD-TVSE), detailing its operative technique and short-term outcomes.

## Case report

A 64-year-old female was admitted with a 1-month history of epigastric pain accompanied by progressive jaundice. Gastroscopy biopsy confirmed moderately differentiated adenocarcinoma in the descending duodenum.

Following general anesthesia, the patient was positioned in the supine lithotomy position. Following standard oncologic resection principles, the distal stomach, entire duodenum, gallbladder, lower common bile duct, pancreatic head, and regional lymph nodes were completely resected. The decision for distal gastrectomy was made to achieve adequate lymphadenectomy around the peripyloric region. The resected specimen was placed into a sterile specimen bag and temporarily retained in the right upper abdominal cavity. Gastrointestinal reconstruction was completed entirely robotically. Pancreaticojejunostomy with mucosa-to-mucosa anastomosis was performed first (Fig. 1A and B). Approximately 10 cm distal to this, a side-to-side choledochojejunostomy was performed (Fig. 1C). Finally, a side-to-side jejunogastrostomy was completed (Fig. 1D). The patient was then repositioned to the reverse Trendelenburg lithotomy position. After vaginal disinfection with povidone-iodine, the posterior vaginal fornix was horizontally incised using an ultrasonic scalpel under robotic visualization (Fig. 2A). A disposable protective sleeve was introduced via the vagina, and the specimen bag was gently guided to the vaginal incision. A sponge forceps was used to grasp one end of the specimen bag, and the specimen, securely contained within the intact specimen bag, was slowly extracted through the vagina (Fig. 2B and C). Subsequently, the posterior vaginal fornix incision was closed using barbed sutures under robotic visualization. Abdominal drainage tubes were placed near the pancreaticojejunostomy and choledochojejunostomy anastomotic sites, respectively.

**Figure 1:**
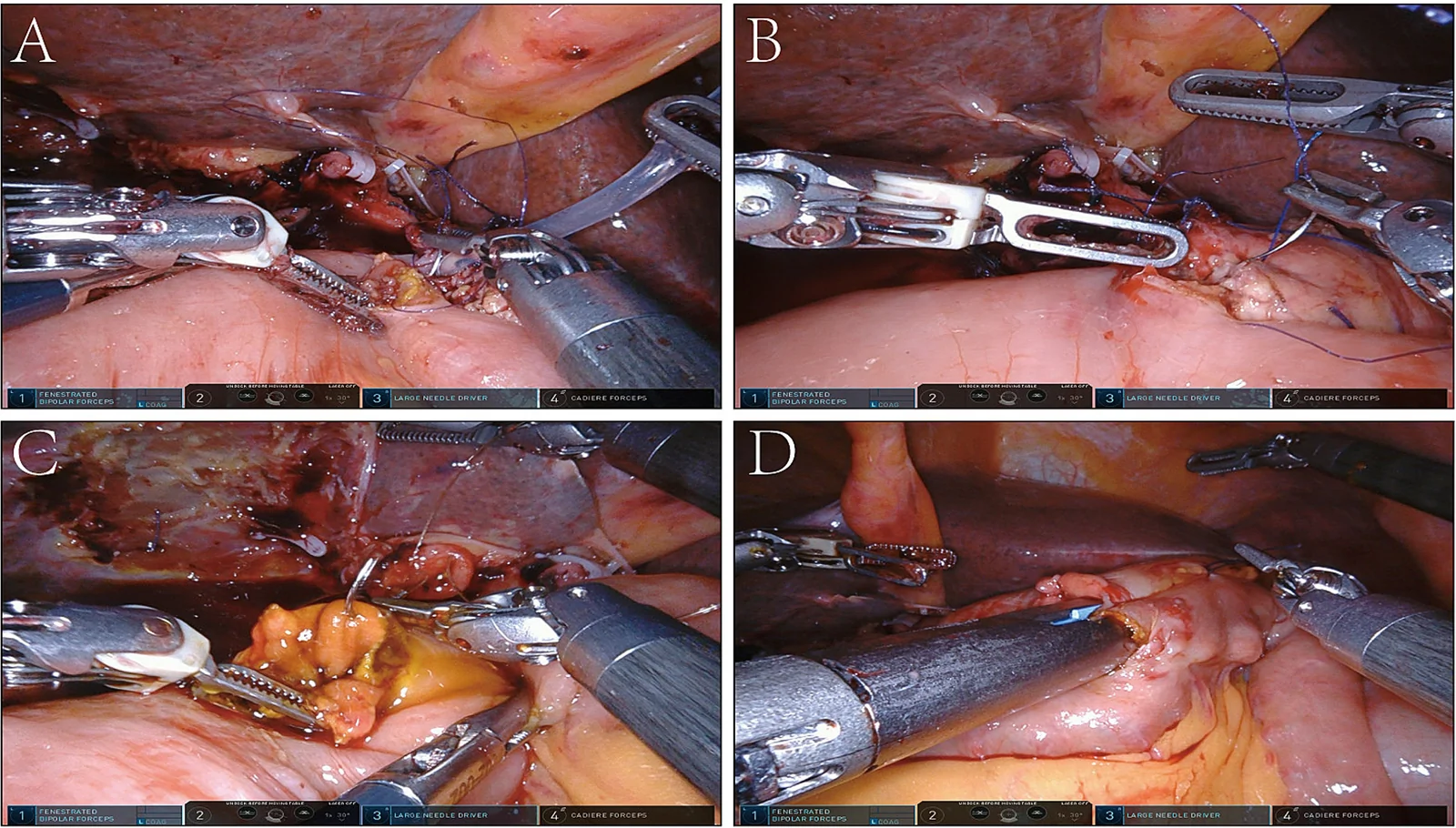
Anastomosis in robotic vision. (A) Duct-mucosa pancreaticojejunostomy. (B) Plasma-muscular layer reinforcement. (C) Bilioenterostomy. (D) Gastroenteric anastomosis.

**Figure 2:**
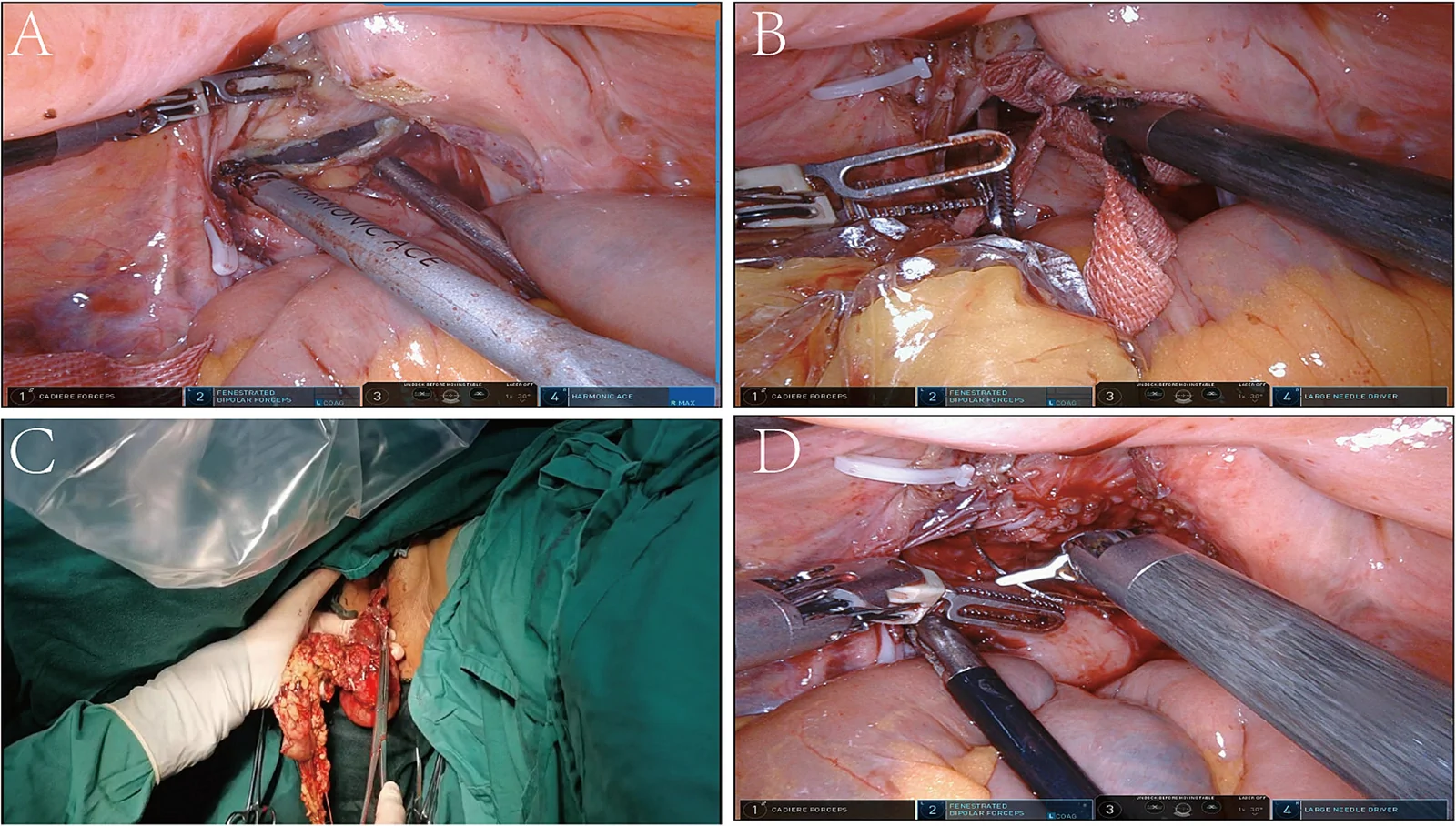
Specimen removal procedure. (A) Posterior fornix of the vagina was cut transverse with ultrasonic knife under the robot vision. (B) Assisted specimens in the abdominal cavity for transvaginal extrication. (C) The specimen is gently extracted transvaginally outside the body. (D) Suture the posterior vaginal fornix with barbed wire under robotic vision.

Postoperative pathology confirmed moderately differentiated adenocarcinoma originating from the duodenal mucosa with a maximum tumor diameter of 2 cm (Fig. 3). Tumor staging was T2N0M0. The patient ambulated on postoperative day 1, and there were no severe perioperative complications. One-year follow-up showed no evidence of recurrence or complications, and no abdominal wall-related complications were observed (Fig. 4B and C).

**Figure 3:**
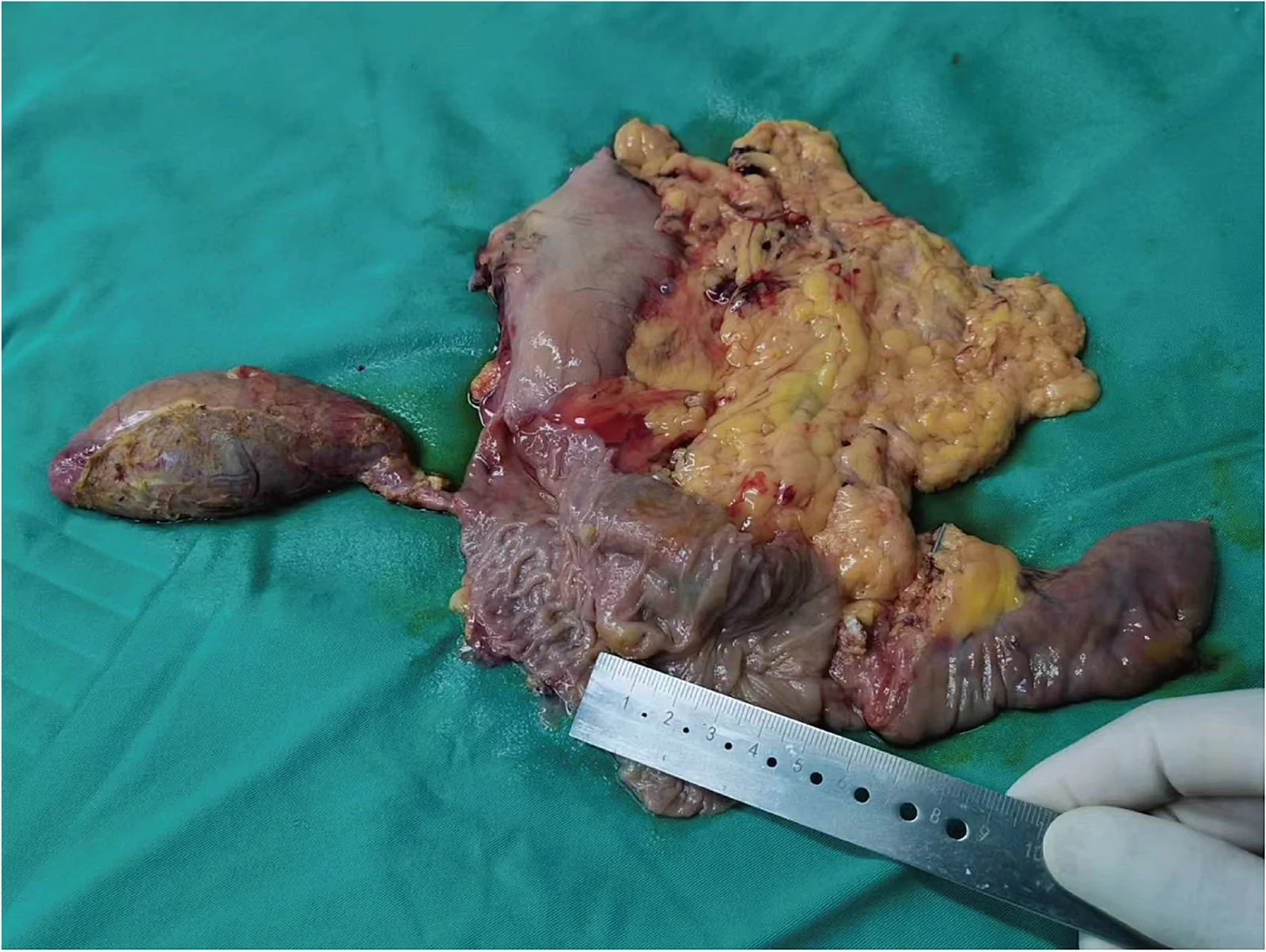
Gross appearance of resected specimen.

**Figure 4:**
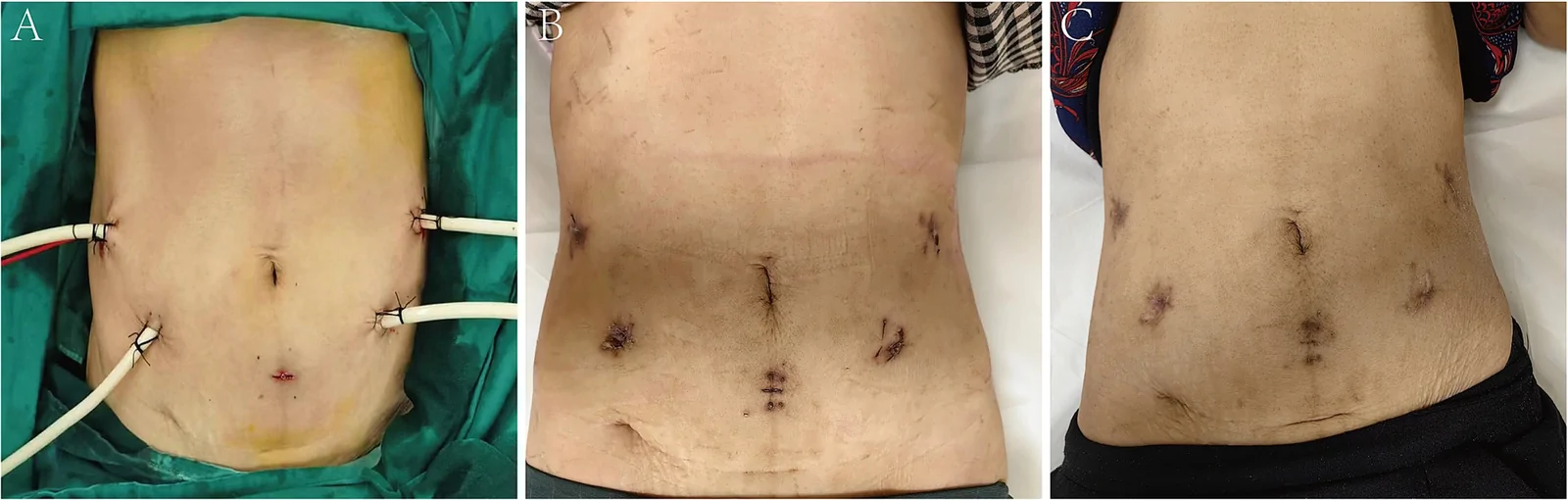
(A) Layout of four abdominal drainage tubes and trocar location. (B) Abdominal condition at discharge. (C) Abdomen at 3-month follow-up after surgery.

## Discussion

One of the primary technical challenges in RPD-TVSE is the transvaginal extraction itself. The posterior vaginal fornix is anatomically adjacent to the rectum, and an imprecise incision risks injury. Furthermore, incision size is critical for safe extraction without tumor rupture. Our specimen's 2 cm diameter allowed for a controlled extraction. Based on this case, we suggest that this technique is most suitable for postmenopausal women with tumors not exceeding 5 cm in maximum diameter, as larger tumors would increase the risk of specimen bag rupture and tumor seeding. No peritoneal contamination or tumor implantation occurred, as the specimen was contained within an intact bag throughout the extraction.

LPD has achieved safety profiles comparable to open surgery [1]. However, the steep learning curve associated with intracorporeal pancreaticojejunostomy has limited its widespread adoption. Robotic systems, equipped with seven degrees of freedom wristed instruments and tremor elimination capabilities, enable more precise anastomotic operations within deep narrow retroperitoneal spaces. Multiple studies have confirmed that RPD yields oncological outcomes comparable to LPD or open PD while demonstrating lower conversion rates, reduced operative times, decreased blood loss, and shortened postoperative hospital stays [5–8]. Furthermore, the incidence of clinically relevant pancreatic fistula is significantly reduced [2, 9].

Compared with traditional transabdominal specimen extraction, the most prominent advantage of NOSES lies in eliminating the need for additional abdominal wall incisions, thereby avoiding incision-related complications such as surgical site infection, wound dehiscence, incisional hernia, and chronic postoperative pain [10]. Zhang *et al.* reported a case series of LPD-TVSE in eight patients, confirming the preliminary safety and feasibility of NOSES in LPD procedures [4]. However, total LPD-TVSE demands exceptional anastomotic skills and vaginal dome manipulation capabilities from surgeons. In comparison, RPD-TVSE combines the precision of robotic anastomosis with the minimally invasive advantages of NOSES. Robotic visualization provides enhanced three-dimensional anatomical detail, which may facilitate precise dissection and incision of the posterior vaginal fornix, potentially reducing the risk of adjacent organ injury [2].

Despite satisfactory short-term outcomes, several limitations warrant consideration. First, patient selection is restricted to female patients, with current recommendations prioritizing postmenopausal women; for premenopausal women desiring fertility preservation, long-term data on pelvic floor function remain unavailable, necessitating cautious patient selection following comprehensive informed consent. In gynecological surgery, transvaginal specimen extraction has been extensively studied, with long-term data indicating no adverse effects on the risk of pelvic infection, sexual function, or subsequent vaginal delivery [11]. Second, equipment dependency and cost concerns: the procedure's heavy reliance on robotic systems entails substantial procurement and maintenance expenses, with overall surgical costs significantly exceeding traditional laparoscopic approaches, limiting adoption in community hospitals. However, some studies suggest RPD may not increase overall hospitalization costs by reducing pancreatitis-related incidence and shortening hospital stay [9]. Third, tumor size constraints: transvaginal specimen extraction imposes volume limitations; patients with large tumors carry potential risks of specimen bag rupture and tumor seeding.

In conclusion, this case successfully demonstrates RPD-TVSE implementation with no perioperative complications and rapid postoperative recovery. Despite current limitations related to patient eligibility and equipment availability, this technique provides a novel minimally invasive treatment option for female perihilar tumors. Larger-scale clinical studies with extended follow-up periods are warranted to further validate this approach's long-term clinical value and oncological safety.
